# Analysis of a Cascaded Piezoelectric Ultrasonic Transducer with Three Sets of Piezoelectric Ceramic Stacks

**DOI:** 10.3390/s19030580

**Published:** 2019-01-30

**Authors:** Xiangdi Meng, Shuyu Lin

**Affiliations:** Shaanxi Key Laboratory of Ultrasonics, Institute of Applied Acoustics, Shaanxi Normal University, Xi’an 710119, China; 13251512317@163.com

**Keywords:** cascaded piezoelectric ultrasonic transducer, longitudinal vibration, resonance frequency, effective electromechanical coupling coefficient

## Abstract

To increase the ultrasonic intensity and power of a piezoelectric transducer, a cascaded piezoelectric ultrasonic transducer with the three sets of piezoelectric ceramic stacks is analyzed. The cascaded piezoelectric ultrasonic transducer consists of four metal cylinders and three sets of piezoelectric ceramic stacks in the longitudinal direction. In analysis, the electromechanical equivalent circuit of the cascaded piezoelectric ultrasonic transducer is obtained, as well as the resonance/anti-resonance frequencies equations. By means of an analytical method, when the position of piezoelectric ceramic stacks PZT-2/PZT-3 changes, the resonance/anti-resonance frequencies and the effective electromechanical coupling coefficient of the cascaded piezoelectric ultrasonic transducer have certain characteristics. Several prototypes of the cascaded piezoelectric ultrasonic transducer are manufactured. The experimentally measured resonance frequencies are in good agreement with the theoretical and simulated results. The cascaded piezoelectric ultrasonic transducer with three sets of piezoelectric ceramic stacks presented in this paper is expected to be used in the field of high power ultrasound.

## 1. Introduction

Piezoelectric ceramic transducers are widely used in power ultrasound fields, due to their small size, light weight, and high efficiency, for applications such as ultrasonic machining, ultrasonic welding, ultrasonic cleaning, ultrasonic cutting, ultrasonic vegetable dehydration, and energy harvesting [1,2,3,4,5,6,7,8]. In general, the most widely used is the sandwich piezoelectric ceramic transducer, also known as the Langevin piezoelectric ceramic transducer. Sandwich piezoelectric transducers have the advantages of high efficiency, good heat dissipation performance, and easy changes of operating frequency and performance parameters [9,10,11,12,13]. Sandwich piezoelectric transducers are valued by researchers.

With the purpose of improving the performance parameters of the sandwich piezoelectric ceramic transducer, substantial studies have been conducted. Athikom et al. designed and analyzed stepped horn sandwich piezoelectric transducers as a particle velocity amplifier [14]. Arnold et al. made a detailed analysis of mechanical pre-stressing on the performance of the piezotransducer [15]. Parrini designed, prototyped, and tested a new high-frequency ultrasonic transducer for wire bonding [16]. Decastro proposed a high-power ultrasonic transducer with broadband frequency for receiving a stimulating signal and producing ultrasound therefrom at one or more frequencies [17]. Lin studied the resonance/anti-resonance frequencies, the effective electromechanical coupling coefficient, and the mechanical quality factor of a sandwich piezoelectric ultrasonic transducer; in addition, a sandwich ultrasonic transducer with two sets of piezoelectric elements was proposed to achieve multi-frequency or wide-frequency bandwidth [18,19]. On the other hand, new structures of the sandwich piezoelectric ceramic transducer have been studied. For example, Zhang et al. and Xu et al. analyzed coupled vibration of composite cylindrical piezoelectric transducers in order to increase the output power and improve the sound radiating efficiency [20,21]. Lin and Xu presented the cascade transducer to simultaneously improve the input electric power and ultrasonic intensity, which was composed of two traditional longitudinally sandwiched piezoelectric transducers [22].

In this paper, in order to further optimize the parameter performance of the transducer, a cascaded piezoelectric ultrasonic transducer with three sets of piezoelectric ceramic stacks is presented. Four metal cylinders and three sets of piezoelectric ceramic stacks are connected together in series mechanically and in parallel electrically. Compared to traditional sandwich piezoelectric ceramic transducers, the cascaded piezoelectric ultrasonic transducer with three sets of piezoelectric ceramic stacks can multiply ultrasonic intensity, ultrasonic power, and heat conductive performance. Due to simultaneous excitation by three sets of piezoelectric ceramic stacks, the cascaded piezoelectric ultrasonic transducer with three sets of piezoelectric ceramic stacks can improve ultrasonic intensity and ultrasonic power better than the cascade transducer, which was composed of two traditional longitudinally sandwiched piezoelectric transducers.

Based on the one-dimensional theory of a transducer, the electromechanical equivalent circuit is obtained first. Then the resonance/anti-resonance frequencies equations are obtained and the resonance/anti-resonance frequencies are studied. Finally, the relationship between the resonance/anti-resonance frequencies and the effective electromechanical coupling coefficient with geometric dimensions is analyzed. Three prototypes of the cascaded piezoelectric ultrasonic transducer are designed to verify the analyses. This provides a theoretical basis for the design and optimization of the transducer.

## 2. Theoretical Analysis

**Figure 1:**
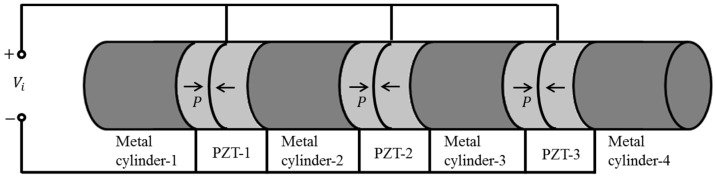
The cascaded piezoelectric ultrasonic transducer with three sets of piezoelectric ceramic stacks.

The cascaded piezoelectric ultrasonic transducer with three sets of piezoelectric ceramic stacks is shown in Figure 1. The arrow P indicates the polarization direction of the piezoelectric ceramic wafer. It consists of four metal cylinders and three sets of longitudinal polarized piezoelectric ceramic stacks. Three sets of longitudinal polarized piezoelectric ceramic stacks are connected together electrically in parallel. Four metal cylinders and three sets of longitudinal polarized piezoelectric ceramic stacks are connected in series. The piezoelectric ceramic stack is composed of two piezoelectric wafers with opposite polarization directions, and piezoelectric wafers are connected by a series of mechanical ends and a parallel connection of electric terminals. A thin metal piece is inserted between the piezoelectric wafer and the piezoelectric wafer, between the piezoelectric wafer and the metal cylinder as an electrode. The metal cylinder and the piezoelectric ceramic stack are connected by high-strength glue or stress bolt. According to the structural features and connection mode of the cascaded piezoelectric ultrasonic transducer, the electrical parallel can multiply ultrasonic power and the mechanical series can multiply ultrasonic intensity. Therefore, the cascaded piezoelectric ultrasonic transducer can simultaneously multiply the input electric power and ultrasonic intensity.

In this paper, the transverse dimension of the transducer is much smaller than the longitudinal vibration dimension, that is, it is much smaller than the longitudinal vibration wavelength. Therefore, the performance of the cascaded piezoelectric transducer can be analyzed by using the one-dimensional theory of transducer.

**Figure 2:**
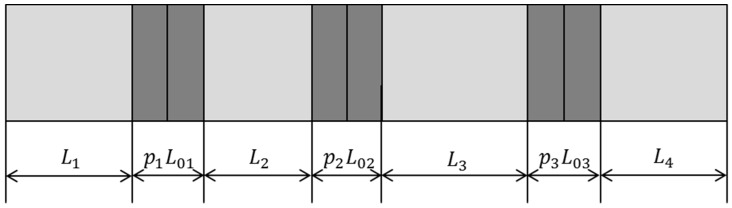
Geometrical diagram of the cascaded piezoelectric ultrasonic transducer with three sets of piezoelectric ceramic stacks.

**Figure 3:**
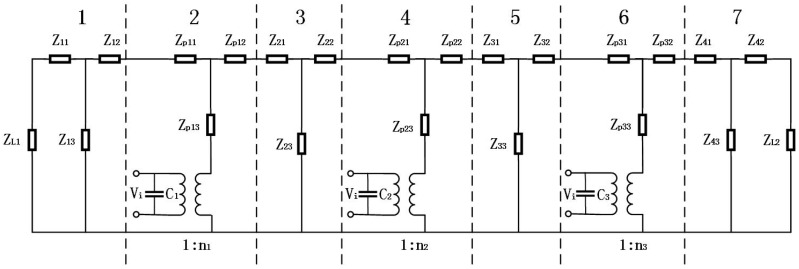
Electromechanical equivalent circuit of the cascaded piezoelectric ultrasonic transducer with three sets of piezoelectric ceramic stacks.

A geometrical diagram of the cascaded piezoelectric ultrasonic transducer with three sets of piezoelectric ceramic stacks is shown in Figure 2. $L_{1}$, $L_{2}$, $L_{3}$, and $L_{4}$ are the length of the four metal cylinders. $p_{1}$, $p_{2}$, $p_{3}$ and $L_{01}$, $L_{02}$, $L_{03}$ are the number of piezoelectric wafers in the three sets of axially polarized piezoelectric ceramic stacks and the length of each piezoelectric wafer. $R_{1}$, $R_{2}$, $R_{3}$, $R_{4}$ and $R_{01}$, $R_{02}$, $R_{03}$ are the radii of the metal cylinder and the piezoelectric ceramic stack, respectively. Based on the one-dimensional theory and the electromechanical equivalent circuit of the sandwich transducer, the equivalent circuit of the cascaded piezoelectric ultrasonic transducer with three sets of piezoelectric ceramic stacks is shown in Figure 3 when mechanical and dielectric losses are not considered.

In Figure 3, parts 1, 3, 5, and 7 represent four metal cylinders, and parts 2, 4, and 6 represent three sets of piezoelectric ceramic stacks, respectively. $V_{i}$ is the input voltage. $Z_{L1}$ and $Z_{L2}$ are the load mechanical impedances. $C_{1}$, $C_{2}$, $C_{3}$ and $n_{1}$, $n_{2}$, $n_{3}$ are the clamped capacitance and the electromechanical transformation coefficient, their expressions are as follows:

(1) $$C_{1}=\frac{p_{1}\varepsilon_{33}^{T}(1-K_{33}^{2})S_{01}}{L_{01}},C_{2}=\frac{p_{2}\varepsilon_{33}^{T}(1-K_{33}^{2})S_{02}}{L_{02}},C_{3}=\frac{p_{3}\varepsilon_{33}^{T}(1-K_{33}^{2})S_{03}}{L_{03}}$$

(2) $$n_{1}=\frac{d_{33}S_{01}}{s_{33}^{E}L_{01}},n_{2}=\frac{d_{33}S_{02}}{s_{33}^{E}L_{02}},n_{3}=\frac{d_{33}S_{03}}{s_{33}^{E}L_{03}},$$

where $\varepsilon_{33}^{T}$, $d_{33}$, $K_{33}$ and $s_{33}^{E}$ are the dielectric constant, the piezoelectric constant, the electromechanical coupling coefficient, and the elastic compliance constant of the piezoelectric material. $S_{01}$, $S_{02}$, $S_{03}$ are cross-sectional areas of three sets of piezoelectric ceramic stacks. $S_{01}=\pi R_{01}^{2}$, $S_{02}=\pi R_{02}^{2}$, $S_{03}=\pi R_{03}^{2}$. $Z_{11}$, $Z_{12}$, $Z_{13}$, $Z_{21}$, $Z_{22}$, $Z_{23}$, $Z_{31}$, $Z_{32}$, $Z_{33}$, $Z_{41}$, $Z_{42}$, $Z_{43}$ and $Z_{p11}$, $Z_{p12}$, $Z_{p13}$, $Z_{p21}$, $Z_{p22}$, $Z_{p23}$, $Z_{p31}$, $Z_{p32}$, $Z_{p33}$ are the series and parallel impedances for four metal cylinders and the three sets of piezoelectric ceramic stacks from left to right; their expressions are as follows:

(3) $$Z_{11}=Z_{12}=jZ_{1}\tan(k_{1}L_{1}/2),Z_{13}=Z_{1}/\left[j\sin(k_{1}L_{1})\right]$$

(4) $$Z_{21}=Z_{22}=jZ_{2}\tan(k_{2}L_{2}/2),Z_{23}=Z_{2}/\left[j\sin(k_{2}L_{2})\right]$$

(5) $$Z_{31}=Z_{32}=jZ_{3}\tan(k_{3}L_{3}/2),Z_{33}=Z_{3}/\left[j\sin(k_{3}L_{3})\right]$$

(6) $$Z_{41}=Z_{42}=jZ_{4}\tan(k_{4}L_{4}/2),Z_{43}=Z_{4}/\left[j\sin(k_{4}L_{4})\right]$$

(7) $$Z_{p11}=Z_{p12}=jZ_{01}\tan(p_{1}k_{0}L_{01}/2),Z_{p13}=Z_{01}/[j\sin(p_{1}k_{0}L_{01})]$$

(8) $$Z_{p21}=Z_{p22}=jZ_{02}\tan(p_{2}k_{0}L_{02}/2),Z_{p23}=Z_{02}/[j\sin(p_{2}k_{0}L_{02})]$$

(9) $$Z_{p31}=Z_{p32}=jZ_{03}\tan(p_{3}k_{0}L_{03}/2),Z_{p33}=Z_{03}/[j\sin(p_{3}k_{0}L_{03})],$$

where $Z_{1}=\rho_{1}c_{1}S_{1}$, $Z_{2}=\rho_{2}c_{2}S_{2}$, $Z_{3}=\rho_{3}c_{3}S_{3}$, $Z_{4}=\rho_{4}c_{4}S_{4}$, $Z_{01}=\rho_{0}c_{0}S_{01}$, $Z_{02}=\rho_{0}c_{0}S_{02}$, $Z_{03}=\rho_{0}c_{0}S_{03}$, $k_{1}=\omega /c_{1}$, $k_{2}=\omega /c_{2}$, $k_{3}=\omega /c_{3}$, $k_{4}=\omega /c_{4}$, $k_{0}=\omega /c_{0}$, $c_{1}={\left(E_{1}/\rho_{1}\right)}^{1/2}$, $c_{2}={\left(E_{2}/\rho_{2}\right)}^{1/2}$, $c_{3}={\left(E_{3}/\rho_{3}\right)}^{1/2}$, $c_{4}={\left(E_{4}/\rho_{4}\right)}^{1/2}$, $c_{0}={\left[1/(s_{33}^{E}\rho_{0})\right]}^{1/2}$, $S_{1}=\pi R_{1}^{2}$, $S_{2}=\pi R_{2}^{2}$, $S_{3}=\pi R_{3}^{2}$, $S_{4}=\pi R_{4}^{2}$. $E_{1}$, $\rho_{1}$, $E_{2}$, $\rho_{2}$, $E_{3}$, $\rho_{3}$ and $E_{4}$, $\rho_{4}$ are the density and Young’s modulus of four metal cylinders. $c_{1}$, $k_{1}$, $c_{2}$, $k_{2}$, $c_{3}$, $k_{3}$, $c_{4}$, $k_{4}$ and $c_{0}$, $k_{0}$ are sound speed and wavenumbers of longitudinal vibration of four metal cylinders and piezoelectric ceramic stacks.

**Figure 4:**
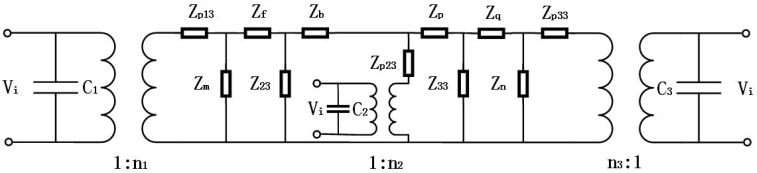
Transformed electromechanical equivalent circuit of the cascaded piezoelectric ultrasonic transducer with three sets of piezoelectric ceramic stacks.

By performing some circuit transformations on the electromechanical equivalent circuit of the cascaded piezoelectric ultrasonic transducer with three sets of piezoelectric ceramic stacks of Figure 3, Figure 4 can be obtained. The expressions of circuit impedances are as follows in Figure 4:

(10) $$Z_{m}=Z_{p11}+Z_{12}+\frac{Z_{13}\left(Z_{11}+Z_{L1}\right)}{Z_{13}+Z_{11}+Z_{L1}}$$

(11) $$Z_{n}=Z_{p32}+Z_{41}+\frac{Z_{43}\left(Z_{42}+Z_{L2}\right)}{Z_{43}+Z_{42}+Z_{L2}}$$

(12) $$Z_{f}=Z_{p12}+Z_{21}$$

(13) $$Z_{b}=Z_{p21}+Z_{22}$$

(14) $$Z_{p}=Z_{p22}+Z_{31}$$

(15) $$Z_{q}=Z_{p31}+Z_{32}.$$

**Figure 5:**
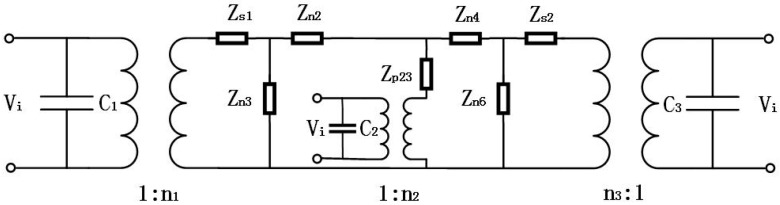
Simplified equivalent circuit of the cascaded piezoelectric ultrasonic transducer with three sets of piezoelectric ceramic stacks.

Figure 5 can be obtained after two more circuit transformations of a star‒triangle‒star circuit. The expressions of circuit impedances are as follows in Figure 5:

(16) $$Z_{s1}=Z_{p13}+Z_{n1}$$

(17) $$Z_{s2}=Z_{n5}+Z_{p33}$$

(18) $$Z_{n1}=\frac{Z_{t1}Z_{m1}}{Z_{t1}+Z_{m1}+Z_{t3}}$$

(19) $$Z_{n2}=\frac{Z_{t1}Z_{t3}}{Z_{t1}+Z_{m1}+Z_{t3}}$$

(20) $$Z_{n3}=\frac{Z_{m1}Z_{t3}}{Z_{t1}+Z_{m1}+Z_{t3}}$$

(21) $$Z_{n4}=\frac{Z_{t4}Z_{t5}}{Z_{t4}+Z_{t5}+Z_{m2}}$$

(22) $$Z_{n5}=\frac{Z_{t4}Z_{m2}}{Z_{t4}+Z_{t5}+Z_{m2}}$$

(23) $$Z_{n6}=\frac{Z_{t5}Z_{m2}}{Z_{t4}+Z_{t5}+Z_{m2}}$$

(24) $$Z_{m1}=\frac{Z_{m}Z_{t2}}{Z_{m}+Z_{t2}}$$

(25) $$Z_{m2}=\frac{Z_{n}Z_{t6}}{Z_{n}+Z_{t6}}$$

(26) $$Z_{t1}=\frac{Z_{f}Z_{b}+Z_{f}Z_{23}+Z_{b}Z_{23}}{Z_{23}}$$

(27) $$Z_{t2}=\frac{Z_{f}Z_{b}+Z_{f}Z_{23}+Z_{b}Z_{23}}{Z_{b}}$$

(28) $$Z_{t3}=\frac{Z_{f}Z_{b}+Z_{f}Z_{23}+Z_{b}Z_{23}}{Z_{f}}$$

(29) $$Z_{t4}=\frac{Z_{p}Z_{q}+Z_{p}Z_{33}+Z_{q}Z_{33}}{Z_{33}}$$

(30) $$Z_{t5}=\frac{Z_{p}Z_{q}+Z_{p}Z_{33}+Z_{q}Z_{33}}{Z_{q}}$$

(31) $$Z_{t6}=\frac{Z_{p}Z_{q}+Z_{p}Z_{33}+Z_{q}Z_{33}}{Z_{p}}.$$

The total input electrical impedance $Z_{i}$ of the cascaded piezoelectric ultrasonic transducer is obtained:

(32) $$Z_{i}=\frac{Z_{i1}Z_{i2}Z_{i3}}{Z_{i1}+Z_{i2}+Z_{i3}}$$

(33) $$Z_{i1}=\frac{Z_{c1}Z_{rm1}}{n_{1}^{2}Z_{c1}+Z_{rm1}}$$

(34) $$Z_{i2}=\frac{Z_{c2}Z_{rm2}}{n_{2}^{2}Z_{c2}+Z_{rm2}}$$

(35) $$Z_{i3}=\frac{Z_{c3}Z_{rm3}}{n_{3}^{2}Z_{c3}+Z_{rm3}}$$

(36) $$Z_{c1}=\frac{1}{j\omega C_{1}}$$

(37) $$Z_{c2}=\frac{1}{j\omega C_{2}}$$

(38) $$Z_{c3}=\frac{1}{j\omega C_{3}}$$

(39) $$Z_{rm1}=\frac{\left\{\begin{array}{l} \left(Z_{s1}Z_{n2}+Z_{s1}Z_{n3}+Z_{n2}Z_{n3}\right)\left[Z_{s2}\left(Z_{n4}+Z_{n6}+Z_{p23}\right)+Z_{n6}\left(Z_{n4}+Z_{p23}\right)\right]\\ +Z_{p23}\left(Z_{s1}+Z_{n3}\right)\left(Z_{s2}Z_{n4}+Z_{s2}Z_{n6}+Z_{n4}Z_{n6}\right) \end{array}\right\}}{\left\{\begin{array}{l} Z_{s2}Z_{n2}\left(Z_{n4}+Z_{n6}+Z_{p23}\right)+Z_{n2}Z_{n6}\left(Z_{p23}+Z_{n4}\right)+Z_{s2}Z_{p23}\left(Z_{n3}+Z_{n4}+Z_{n6}\right)\\ +Z_{n3}\left(Z_{s2}Z_{n6}+Z_{s2}Z_{n4}+Z_{n4}Z_{n6}\right)\left(1-\frac{n_{2}}{n_{1}}\right)+Z_{n3}Z_{n6}Z_{p23}\left(1-\frac{n_{3}}{n_{1}}\right)+Z_{n4}Z_{n6}Z_{p23} \end{array}\right\}}$$

(40) $$Z_{rm2}=\frac{\left\{\begin{array}{l} \left(Z_{s1}Z_{n2}+Z_{s1}Z_{n3}+Z_{n2}Z_{n3}\right)\left[Z_{s2}\left(Z_{n4}+Z_{n6}+Z_{p23}\right)+Z_{n6}\left(Z_{n4}+Z_{p23}\right)\right]\\ +Z_{p23}\left(Z_{s1}+Z_{n3}\right)\left(Z_{s2}Z_{n4}+Z_{s2}Z_{n6}+Z_{n4}Z_{n6}\right) \end{array}\right\}}{\left\{\begin{array}{l} Z_{s1}Z_{s2}\left(Z_{n2}+Z_{n3}+Z_{n4}+Z_{n6}\right)+Z_{n6}\left(Z_{s1}Z_{n2}+Z_{s1}Z_{n3}+Z_{n2}Z_{n3}\right)\left(1-\frac{n_{3}}{n_{2}}\right)\\ +Z_{n3}\left(Z_{s2}Z_{n4}+Z_{s2}Z_{n6}+Z_{n4}Z_{n6}\right)\left(1-\frac{n_{1}}{n_{2}}\right)+Z_{s1}Z_{n4}Z_{n6}+Z_{s2}Z_{n2}Z_{n3} \end{array}\right\}}$$

(41) $$Z_{rm3}=\frac{\left\{\begin{array}{l} \left(Z_{s1}Z_{n2}+Z_{s1}Z_{n3}+Z_{n2}Z_{n3}\right)\left[Z_{s2}\left(Z_{n4}+Z_{n6}+Z_{p23}\right)+Z_{n6}\left(Z_{n4}+Z_{p23}\right)\right]\\ +Z_{p23}\left(Z_{s1}+Z_{n3}\right)\left(Z_{s2}Z_{n4}+Z_{s2}Z_{n6}+Z_{n4}Z_{n6}\right) \end{array}\right\}}{\left\{\begin{array}{l} Z_{n6}\left(Z_{s1}Z_{n2}+Z_{s1}Z_{n3}+Z_{n2}Z_{n3}\right)\left(1-\frac{n_{2}}{n_{3}}\right)+Z_{n3}Z_{n6}Z_{p23}\left(1-\frac{n_{1}}{n_{3}}\right)\\ +\left(Z_{s1}Z_{n2}+Z_{s1}Z_{n3}+Z_{n2}Z_{n3}\right)\left(Z_{n4}+Z_{n6}\right)+Z_{p23}\left(Z_{s1}Z_{n6}+Z_{s1}Z_{n4}+Z_{n3}Z_{n4}\right) \end{array}\right\}},$$

where $Z_{i1}$, $Z_{i2}$, $Z_{i3}$ and $Z_{rm1}$, $Z_{rm2}$, $Z_{rm3}$ are the input impedances and the mechanical impedances of the three sets of piezoelectric ceramic stacks. $Z_{c1}$, $Z_{c2}$, $Z_{c3}$ are the impedances of the clamped capacitances.

According to the definition of the resonance/anti-resonance frequencies, the resonance/anti-resonance frequencies equations are obtained:

(42) $$Z_{i}=0$$

(43) $$Z_{i}\to \infty .$$

The resonance frequency equation is important for the transducer engineering design, and at the same time, it is the basis for the transducer analysis. From Equations (42) and (43), when the material parameters and geometric dimensions of the cascaded piezoelectric ultrasonic transducer with three sets of piezoelectric ceramic stacks are given, the effective electromechanical coupling coefficient can is calculated:

(44) $$K_{eff}=\sqrt{1-{\left(\frac{f_{r}}{f_{a}}\right)}^{2}}.$$

**Figure 6:**
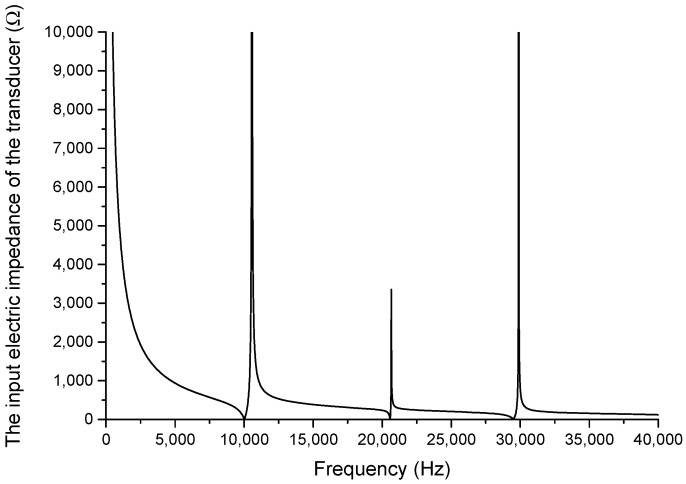
The frequency curve of the input electrical impedance of the cascaded piezoelectric ultrasonic transducer.

From Equation (32), when the transducer is under no-load condition, the frequency curve of the input electrical impedance is obtained in Figure 6. Aluminum and PZT-4 are materials for the metal cylinders and the piezoelectric ceramic stacks. The material parameters are as follows: $\rho_{1}=\rho_{2}=\rho_{3}=2700\;\mathrm{kg}/m^{3}$, $E_{1}=E_{2}=E_{3}=7.023\times 10^{10}\;N/\mu^{2}$, $\rho_{0}=7500\;\mathrm{kg}/m^{3}$, $s_{33}^{E}=15.5\times 10^{-12}\;\mu^{2}/N$, $\varepsilon_{33}^{T}/\varepsilon_{0}=1300$, $K_{33}=0.7$, $d_{33}=496\times 10^{-12}\;C/N$, $\varepsilon_{0}=8.8542\times 10^{-12}\;F/m$. The geometric dimensions for the cascaded piezoelectric ultrasonic transducer are designed and exhibited as follows: $p_{1}=p_{2}=2$, $L_{1}=0.03\;m$, $L_{2}=0.04\;m$, $L_{3}=0.07\;m$, $L_{4}=0.06\;m$, $L_{01}=L_{02}=L_{03}=0.005\;m$, $R_{1}=R_{2}=R_{3}=R_{4}=R_{01}=R_{02}=R_{03}=0.02\;m$.

In Figure 6, the x-axis represents the frequency and the y-axis represents the input impedance of the cascaded piezoelectric ultrasonic transducer. When mechanical losses, dielectric losses, and loads are neglected, it can be seen from Figure 6 that, on the one hand, the minimum value of the input electrical impedance is equal to zero and the maximum value of the input electrical impedance is infinite; on the other hand, the cascaded piezoelectric ultrasonic transducer has multiple resonance/anti-resonance frequencies, which means the transducer has multiple vibrational modes. The vibrational characteristics of the cascaded piezoelectric ultrasonic transducer are different in different vibrational modes. Different modes can be used in different applications.

## 3. Theoretical Relationships between Performance Parameters and Geometric Dimensions of the Cascaded Piezoelectric Ultrasonic Transducer

The relationships between the resonance frequency/anti-resonance frequencies and the effective electromechanical coupling coefficient with geometric dimensions are theoretically studied in order to analyze the electromechanical characteristics of the cascaded piezoelectric ultrasonic transducer with three sets of piezoelectric ceramic stacks. Based on Equations (42) and (43), the resonance/anti-resonance frequencies are first obtained, and then the effective electromechanical coupling coefficient is calculated by Equation (44). In this section, the metal cylinders and PZT-4 are chosen as aluminum and the piezoelectric material. The material parameters are the same as those in Section 2. Since the transducer contains three sets of piezoelectric ceramic stacks, when the total size of the transducer is kept constant and the positions of the two sets of piezoelectric ceramic stacks are fixed, change in distance between one set of piezoelectric ceramic stacks and two other sets of piezoelectric ceramic stacks can be realized by changing the length of the metal cylinder. Position changes of piezoelectric ceramic stacks PZT-2 and PZT-3 are studied.

### 3.1. Position Effect of Piezoelectric Ceramic Stacks PZT-2

**Figure 7:**
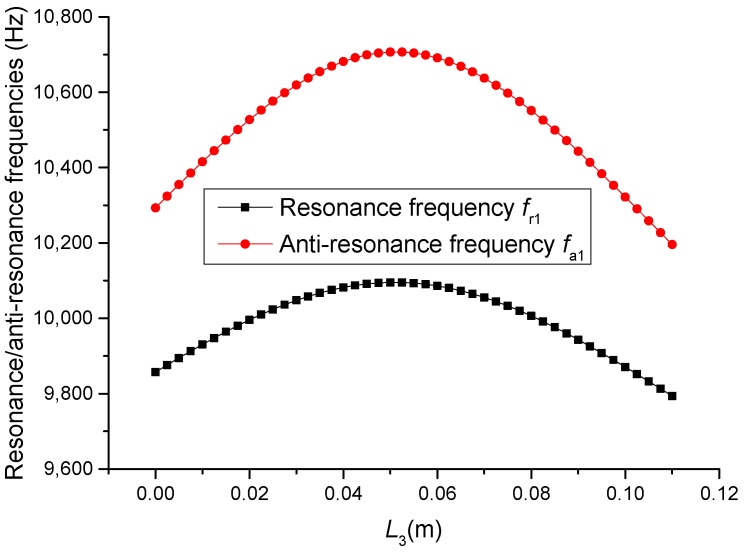
Theoretical relationships between the resonance/anti-resonance frequencies and length $L_{3}$ at the fundamental mode (Position effect of PZT-2).

**Figure 8:**
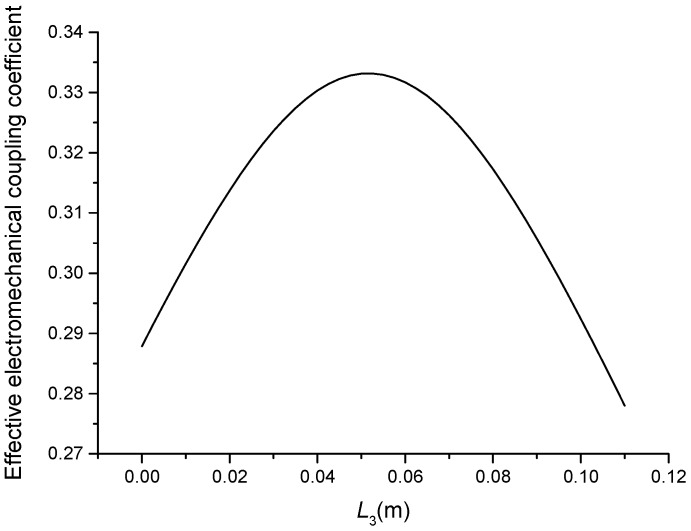
Theoretical relationship between the effective electromechanical coupling coefficient and length $L_{3}$ at the fundamental mode (Position effect of PZT-2).

**Figure 9:**
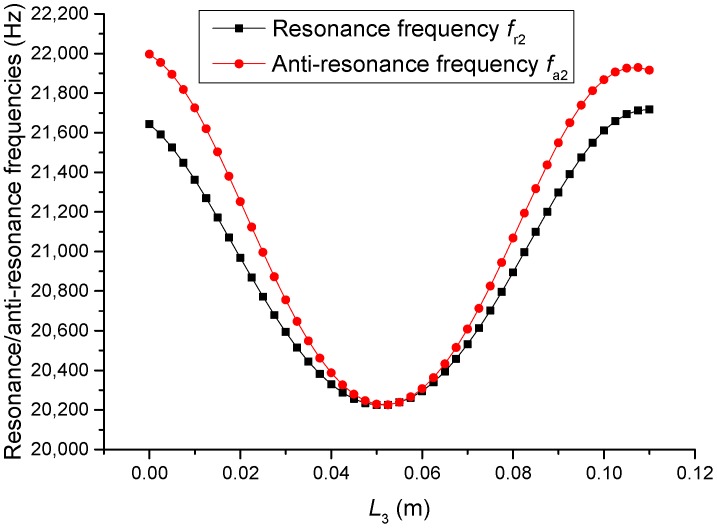
Theoretical relationships between the resonance/anti-resonance frequencies and length $L_{3}$ at the second mode (Position effect of PZT-2).

**Figure 10:**
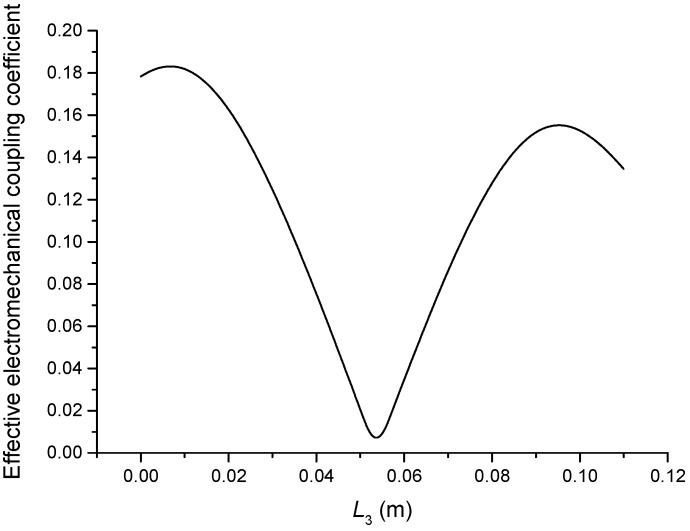
Theoretical relationship between the effective electromechanical coupling coefficient and length $L_{3}$ at the second mode (Position effect of PZT-2).

The geometric dimensions are $p_{1}=p_{2}=2$, $L_{1}=0.04\;m$, $L_{2}+L_{3}=0.11\;m$, $L_{4}=0.05\;m$, $L_{01}=L_{02}=L_{03}=0.005\;m$, $R_{1}=R_{2}=R_{3}=R_{4}=R_{01}=R_{02}=R_{03}=0.02\;m$. When the length $L_{3}$ is changed, that is, the position of piezoelectric ceramic stacks PZT-2 is changed, the theoretical relationships between the resonance/anti-resonance frequencies, the effective electromechanical coupling coefficient and the length $L_{3}$ are displayed in Figure 7, Figure 8, Figure 9 and Figure 10.

As can be seen from Figure 7 and Figure 8, the performance parameters of the cascaded piezoelectric ultrasonic transducer change when the position of piezoelectric ceramic stacks PZT-2 changes at the fundamental mode. When the length $L_{3}$ is increased, the resonance/anti-resonance frequencies and the effective electromechanical coupling coefficient are significantly increased. However, when the PZT-2 is in the middle position, there is a maximum value for the resonance/anti-resonance frequencies and the effective electromechanical coupling coefficient, which is the design goal of the transducer. As can be seen from Figure 9 and Figure 10, when the PZT-2 is in a certain position, the resonance/anti-resonance frequencies and the effective electromechanical coupling coefficient appear to be a minimum value at the second mode. There is avoided in the design process of the transducer.

### 3.2. Position Effect of Piezoelectric Ceramic Stacks PZT-3

**Figure 11:**
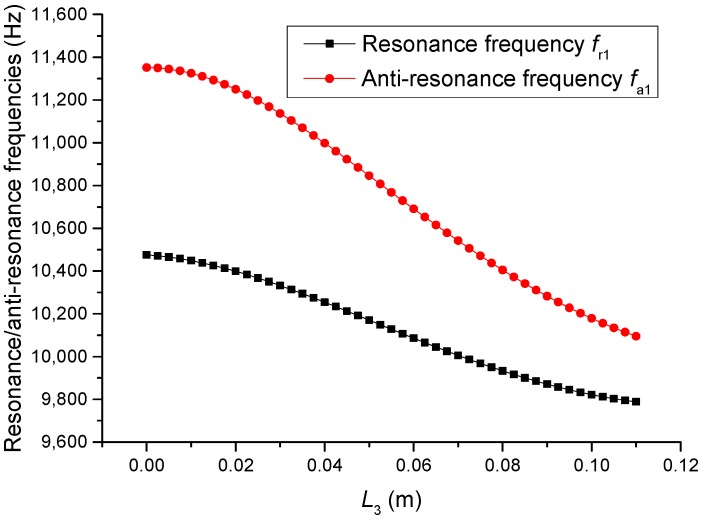
Theoretical relationships between the resonance/anti-resonance frequencies and length $L_{3}$ at the fundamental mode (Position effect of PZT-3).

**Figure 12:**
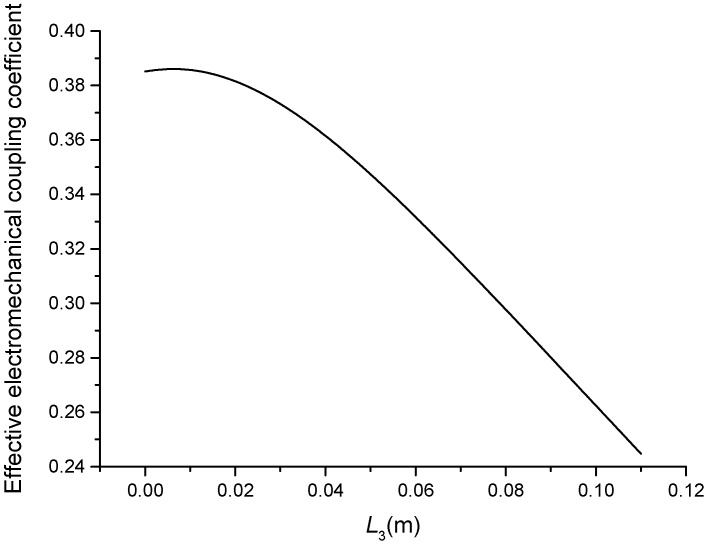
Theoretical relationship between the effective electromechanical coupling coefficient and length $L_{3}$ at the fundamental mode (Position effect of PZT-3).

**Figure 13:**
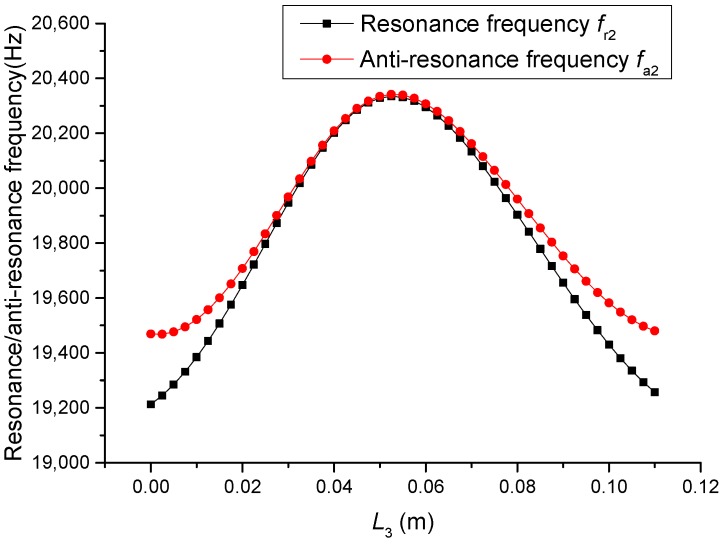
Theoretical relationships between the resonance/anti-resonance frequencies and length $L_{3}$ at the second mode (Position effect of PZT-3).

**Figure 14:**
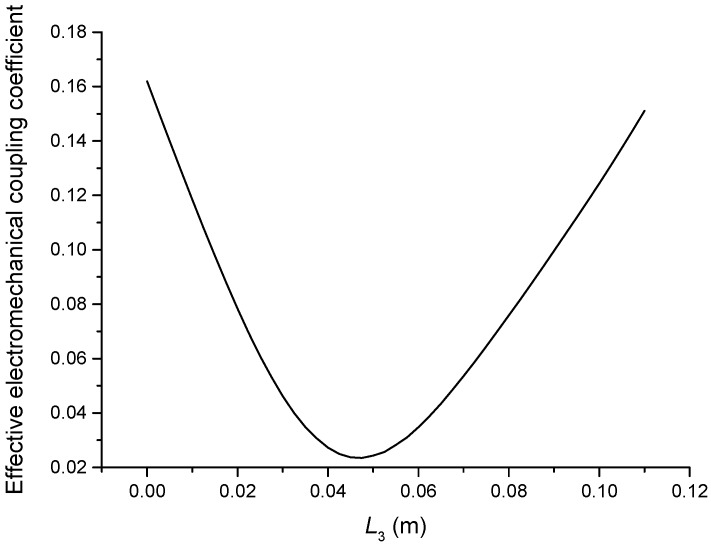
Theoretical relationship between the effective electromechanical coupling coefficient and length $L_{3}$ at the second mode (Position effect of PZT-3).

The geometric dimensions are $p_{1}=p_{2}=2$, $L_{1}=0.04\;m$, $L_{2}=0.05\;m$, $L_{3}+L_{4}=0.11\;m$, $L_{01}=L_{02}=L_{03}=0.005\;m$, $R_{1}=R_{2}=R_{3}=R_{4}=R_{01}=R_{02}=R_{03}=0.02\;m$. When the length $L_{3}$ is changed, that is, the position of piezoelectric ceramic stacks PZT-3 is changed, the theoretical relationships between the resonance/anti-resonance frequencies, the effective electromechanical coupling coefficient and the length $L_{3}$ are displayed in Figure 11, Figure 12, Figure 13 and Figure 14. 

As can be seen from Figure 11 and Figure 12, the performance parameters of the cascaded piezoelectric ultrasonic transducer change when the position of piezoelectric ceramic stacks PZT-3 changes at the fundamental mode. When the length $L_{3}$ is increased, the resonance/anti-resonance frequencies and the effective electromechanical coupling coefficient are gradually decreased. As can be seen from Figure 13 and Figure 14, when the PZT-3 is in a certain position, the resonance/anti-resonance frequencies appear to be a maximum value and the effective electromechanical coupling coefficient appear to be a minimum value at the second mode.

## 4. Experimental

In Section 3, the resonance/anti-resonance frequencies and the effective electromechanical coupling coefficient are calculated. Experimental validation is carried out in this section. The geometrical dimensions are designed and listed in Table 1. The materials for three cascaded piezoelectric ultrasonic transducers are the same as those in Section 2 and Section 3.

**Figure 15:**
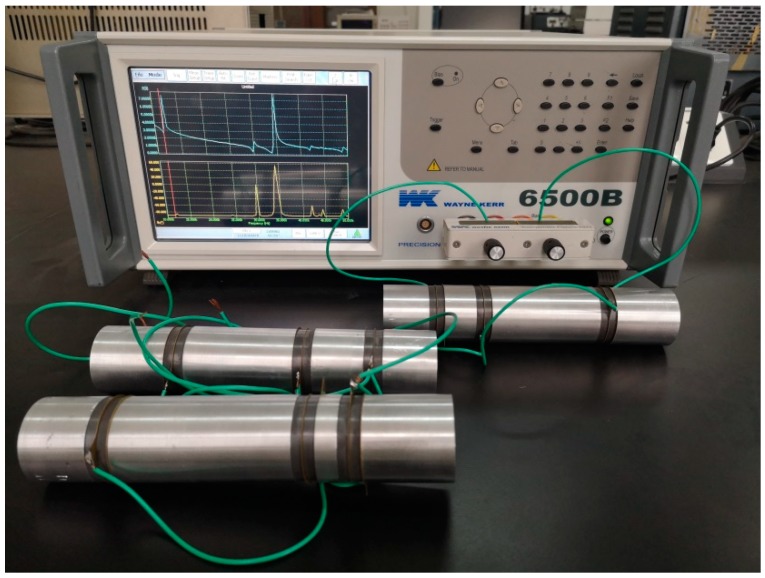
Experimental setup for the measurement of the resonance/anti-resonance frequencies.

**Figure 16:**
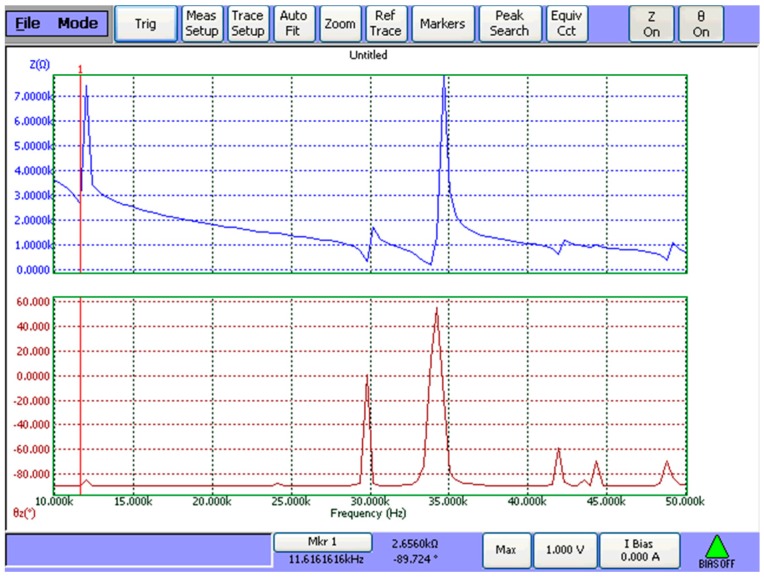
Measured frequency response of the input electric impedance for No.1 cascaded piezoelectric ultrasonic transducer.

Based on Equations (42) and (43), the resonance/anti-resonance frequencies of three cascaded transducers are obtained. The theoretical results are listed in Table 2 and Table 3 by using Wolfram Mathematica 9.0. The resonance/anti-resonance frequencies are experimentally tested by WK6500B Precision Impedance Analyzer, as shown in Figure 15. The measured magnitude and phase of input electric impedance curves of No. 1 cascaded piezoelectric ultrasonic transducer are displayed in Figure 16. The measured resonance/anti-resonance frequencies at the fundamental and second mode are listed in Table 2 and Table 3.

**Figure 17:**
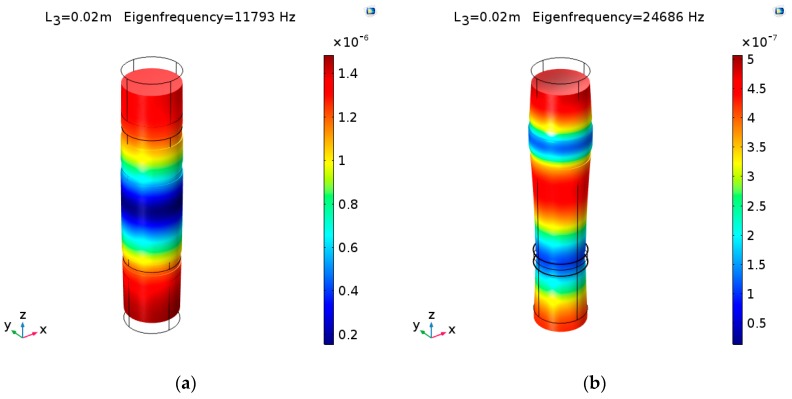
The vibration modes of No. 1 cascaded piezoelectric ultrasonic transducer: (**a**) fundamental mode; (**b**) second mode.

On the other hand, models of three cascaded transducers are established using COMSOL Multiphysics 5.3. The materials for three cascaded piezoelectric ultrasonic transducers are the same as before. The vibration modes of No. 1 cascaded piezoelectric ultrasonic transducer at the fundamental and second mode are shown in Figure 17 ((a) and (b) are No. 1 cascaded piezoelectric ultrasonic transducer at the fundamental and second mode, respectively). The simulated resonance/anti-resonance frequencies are listed in Table 2 and Table 3. In these tables, $f_{r}$, $f_{a}$, $f_{rn}$, $f_{an}$ and $f_{rm}$, $f_{am}$ are the theoretical, simulated and experimental resonance/anti-resonance frequencies. 

The theoretical, simulated, and experimental effective electromechanical coupling coefficients of three cascaded transducers are calculated by Equation (44) and listed in Table 4. In the table, $K_{eff}$, $K_{eff-n}$ and $K_{eff-m}$ are the theoretical, simulated, and experimental effective electromechanical coupling coefficient.

From Table 2, Table 3 and Table 4, it can be seen that the resonance/anti-resonance frequencies from the one-dimensional theory are in good agreement with the numerical simulated and experimental results; the theoretical, simulated, and experimental effective electromechanical coupling coefficients are essentially consistent with each other. The main reasons for the errors are as follows: (1) The standard material parameters in the theoretical analysis deviate from the real material parameters; (2) The mechanical loss and the dielectric loss are ignored in theoretical analysis; (3) In theoretical analysis, the prestressing bolt is ignored, but the transducers are clamped by a prestressing metal bolt in the experiments.

## 5. Conclusions

The cascaded piezoelectric ultrasonic transducer with three sets of piezoelectric ceramic stacks is studied. Based on one-dimensional longitudinal vibration theory, the resonance/anti-resonance frequency equations are obtained. Position effects of piezoelectric ceramic stacks PZT-2 and PZT-3 are analyzed. In summary, the following conclusions can be obtained.

(1) When the position of piezoelectric ceramic stacks PZT-2 changes, for the fundamental mode, the resonance/anti-resonance frequencies and the effective electromechanical coupling coefficient of the cascaded piezoelectric ultrasonic transducer have maximum values; for the second mode, the resonance/anti-resonance frequencies and the effective electromechanical coupling coefficient have minimum values.

(2) When the position of piezoelectric ceramic stacks PZT-3 changes, for the fundamental mode, when PZT-3 is far away from PZT-2, the resonance/anti-resonance frequencies and the effective electromechanical coupling coefficient are gradually decreased; for the second mode, the resonance/anti-resonance frequencies have maximum values and the effective electromechanical coupling coefficient has a minimum value.

(3) By properly choosing the position of piezoelectric ceramic stacks, the performance of the cascaded piezoelectric ultrasonic transducer can be optimized.

(4) The theoretical resonance/anti-resonance frequencies and effective electromechanical coupling coefficients are in good agreement with the simulated and experimental results. 

## Figures and Tables

**Table 1:** Geometrical dimensions for the cascaded piezoelectric ultrasonic transducer.

**No.**	***L*_1_ (mm)**	***L*_2_ (mm)**	***L*_3_ (mm)**	***L*_4_ (mm)**	***L*_01_ = *L*_02_ = *L*_03_ (mm)**
**1**	40.0	70.0	20.0	30.0	5.0
**2**	40.0	60.0	30.0	30.0	5.0
**3**	40.0	10.0	80.0	30.0	5.0
**No.**	***p*_1_ = *p*_2_ = 2**		***R*_1_ = *R*_2_ = *R*_3_ = *R*_4_ (mm)**		***R*_01_ = *R*_02_ = *R*_03_ (mm)**
**1**	2.0		19.5		19.0
**2**	2.0		19.5		19.0
**3**	2.0		19.5		19.0

**Table 2:** The theoretical, simulated and experimental resonance/anti-resonance frequencies of the transducers at the fundamental mode.

No.	Theoretical Results		Simulated Results		Experimental Results	
	$f_{r}\;(\mathrm{Hz})$	$f_{a}\;(\mathrm{Hz})$	$f_{rn}\;(\mathrm{Hz})$	$f_{an}\;(\mathrm{Hz})$	$f_{rm}\;(\mathrm{Hz})$	$f_{am}\;(\mathrm{Hz})$
**1**	11847	12608	11792	12278	11703	11935
**2**	11929	12765	11874	12405	11935	12136
**3**	11829	12561	11775	12247	11751	12006

**Table 3:** The theoretical, simulated, and experimental resonance/anti-resonance frequencies of the transducers at the second mode.

No.	Theoretical Results		Simulated Results		Experimental Results	
	$f_{r}\;(\mathrm{Hz})$	$f_{a}\;(\mathrm{Hz})$	$f_{rn}\;(\mathrm{Hz})$	$f_{an}\;(\mathrm{Hz})$	$f_{rm}\;(\mathrm{Hz})$	$f_{am}\;(\mathrm{Hz})$
**1**	25118	25439	24682	24888	23747	24218
**2**	24537	24693	24156	24253	23240	23327
**3**	25241	25788	24793	25147	23896	24154

**Table 4:** The theoretical, simulated, and experimental effective electromechanical coupling coefficients of the transducers at the fundamental and second mode.

No.	The Fundamental Mode			The Second Mode		
	$K_{eff}$	$K_{eff-n}$	$K_{eff-m}$	$K_{eff}$	$K_{eff-n}$	$K_{eff-m}$
**1**	0.342	0.279	0.196	0.159	0.128	0.196
**2**	0.356	0.289	0.181	0.112	0.089	0.086
**3**	0.336	0.275	0.205	0.205	0.167	0.146
